# Inter-professional agreement and collaboration between extended scope physiotherapists and orthopaedic surgeons in an orthopaedic outpatient shoulder clinic – a mixed methods study

**DOI:** 10.1186/s12891-020-03831-z

**Published:** 2021-01-04

**Authors:** Merete Nørgaard Madsen, Maria Lange Kirkegaard, Thomas Martin Klebe, Charlotte Lorenzen Linnebjerg, Søren Martin Riis Villumsen, Stine Junge Due, Jeanette Trøstrup, Camilla Blach Rossen, Hans Okkels Birk, Brian Elmengaard, Lone Ramer Mikkelsen

**Affiliations:** 1 Elective Surgery Centre, Silkeborg Regional Hospital, Silkeborg, Denmark; 2 Department of Public Health, University of Copenhagen, Copenhagen, Denmark; 3 Department of Clinical Medicine, Aarhus University, Aarhus, Denmark

**Keywords:** Advanced practice, Physiotherapy, Orthopaedics, Shoulder, Diagnosis, Agreement, Coordination, Collaboration

## Abstract

**Background:**

Extended scope physiotherapists (ESP) are increasingly supplementing orthopaedic surgeons (OS) in diagnosing patients with musculoskeletal disorders. Studies have reported satisfactory diagnostic and treatment agreement between ESPs and OSs, but methodological study quality is generally low, and only few studies have evaluated inter-professional collaboration. Our aims were: 1) to evaluate agreement on diagnosis and treatment plan between ESPs and OSs examining patients with shoulder disorders, 2) to explore and evaluate their inter-professional collaboration.

**Methods:**

1. In an orthopaedic outpatient shoulder clinic, 69 patients were examined independently twice on the same day by an ESP and an OS in random order. Primary and secondary diagnoses (nine categories) and treatment plan (five categories, combinations allowed) were registered by each professional and compared. Percentage of agreement and kappa-values were calculated.
2. Two semi-structured focus-group interviews were performed with ESPs and OSs, respectively. Interviews were based on the theoretical concept of Relational Coordination, encompassing seven dimensions of communication and relationship among professionals. A thematic analysis was conducted.

**Results:**

1. Agreement on primary diagnosis was 62% (95% CI: [50; 73]). ESPs and OSs agreed on the combination of diagnoses in 79% (95% CI: [70; 89]) of the cases. Partial diagnostic agreement (one professional’s primary diagnosis was also registered as either primary or secondary diagnosis by the other) was 96% (95% CI: [91; 100]). Across treatment categories, agreement varied between 68% (95% CI: [57; 79]) and 100%. In 43% (95% CI: [31; 54]) of the cases, ESP and OS had full concordance between treatment categories chosen, while they agreed on at least one recommendation in 96% (95% CI: [91; 100]).
2. Positive statements of all dimensions of relational coordination were found. Three themes especially important in the inter-professional collaboration emerged: Close communication, equal and respectful relationship and professional skills.

**Conclusions:**

In the majority of cases, the ESP and OS registered the same or partly the same diagnosis and treatment plan. Indications of a high relational coordination implying a good inter-professional collaboration were found. Our results support that ESPs and OSs can share the task of examining selected patients with shoulder disorders in an orthopaedic clinic.

**Trial registration:**

ClinicalTrials.gov Identifier: NCT03343951. Registered 10 November 2017

**Supplementary Information:**

The online version contains supplementary material available at 10.1186/s12891-020-03831-z.

## Background

Worldwide, musculoskeletal conditions (MSC) are a leading contributor to disability [1] and constitute a major burden to individuals and society [2, 3]. MSC comprise more than 150 diagnoses, including shoulder disorders. Shoulder disorders are broadly characterized by pain and functional limitations [3, 4] and lifetime prevalence of shoulder pain is 7–67% [4]. In general, MSC are managed by different health professionals in both primary and specialist care, with orthopaedic surgeons (OS) being the most commonly consulted type of specialist [5]. However, many patients with MSC, including patients with shoulder disorders, do not need a surgical intervention [6–12]. Thus, they could potentially be managed by a physiotherapist with special training (extended scope physiotherapist, ESP) instead. The use of ESP to examine and diagnose patients with MSC has the potential to reduce patient wait time [7, 11, 13–15], reduce health care costs [9, 13, 16] and contribute to counteract a potential shortage of orthopaedic workforce [10, 17, 18]. Thus, the use of ESPs has increased [9, 19] and is implemented in several countries worldwide [7, 9, 11, 15, 20]. However, when such a practice is implemented, it is important to ensure maintenance of professional, organizational and patient perceived quality of the service provided.

Previous studies, comparing ESPs and OSs examining patients with MSC, have reported satisfactory results regarding agreement on diagnosis and treatment plan, costs and patient satisfaction [12, 21]. As an indicator of professional quality, a diagnostic agreement between ESP and OS ranging from 65 to 98% [15, 22, 23], and agreement on treatment plan from 74 to 97% [14, 15, 22, 24] have been reported. Nevertheless, no conclusive evidence is present because, generally, the methodological quality of the available inter-rater agreement studies is reported to be moderate to low [12, 21]. In the systematic review by Trøstrup et al., 67% (8/12) of the included diagnostic agreement studies were of moderate to low quality, while in the systematic review by Marks et al., all six included agreement studies were reported to be of moderate quality. Hence, further high quality studies comparing ESPs and OSs are needed [12, 21]. This is particularly the case, when it comes to patients with shoulder disorders, as only few studies have exclusively focused on this patient group [15, 23]. A retrospective study reported 65% diagnostic agreement between ESP and OS, but the population only included patients, that ESPs deemed necessary to be examined by an OS as well [23]. Also, some of the patients had an MRI between the examinations, which means ESPs and OSs had different levels of information available when diagnosing [23]. Thus, the generalizability of the results is limited. Another study reported a high agreement both on diagnosis (84–98%) and on indication for surgery (88%) [15]. However, patient history was obtained by the ESP using a standardized questionnaire, which was then shared with the OS. Hence, the examinations were not fully independent. Also, it is unclear whether the order of examinations varied and/or if the participants were blinded to the findings in the individual examinations.

According to organizational factors, in general, studies involving health professionals and health care interventions indicate that inter-professional collaboration influences the quality of care of the patients [25–29]. Thus, the inter-professional collaboration and relation between ESPs and OSs may influence the quality of the shoulder examinations as well. Only few studies have evaluated collaboration and the ESPs’ experiences of managing their task in orthopaedic clinics [19, 30]. Results from a descriptive survey, on ESPs managing patients with back pain, showed that 74% of ESPs found their job stressful [19]. A theme emerging was the need for mutual support and cooperation between OS and ESP [19]. Likewise, a qualitative study describing the experiences of ESPs in four orthopaedic clinics, highlighted the importance of a good relationship with the consultant for the ESP to be satisfied in the position and to achieve success [30]. However, none of the studies interpreted the experiences of the ESPs in the light of associated factors of quality, such as agreement between the ESP and OS on diagnosis and/or treatment. To our knowledge, no mixed-method studies have evaluated the quality of ESPs managing patients with shoulder disorders in terms of investigating diagnostic agreement between ESPs and OSs and exploring the inter-professional collaboration during this task. Such studies allow a deeper understanding of the quality of the shoulder examinations performed by ESPs as a supplement to OSs. Based on existing evidence, there is a need for high quality studies evaluating professional and organizational quality of ESPs managing patients with shoulder disorders.

## Methods

### Study aims

- 1a) To evaluate agreement on diagnosis between ESPs and OSs examining patients with shoulder disorders
- 1b) To evaluate agreement on treatment plan between ESPs and OSs examining patients with shoulder disorders (primary aim)
- 2) To explore and evaluate the inter-professional collaboration between ESPs and OSs

### Setting

At the orthopaedic outpatient shoulder clinic at Silkeborg Regional Hospital (The Shoulder Clinic), the ESPs and OSs share the task of examining patients referred from general practitioners to the hospital for a specialist evaluation. The initiative to share this task came from both professions, based on the intention to optimize the use of both professions’ skills in order to provide the best quality of care for the patients. The concept gradually evolved from 2009 and onwards to the current clinical practice (Table 1). During the process, a specialist education program was developed (Table 2) and had to be completed by the physiotherapist before becoming eligible to work as an ESP. Furthermore, the distribution of roles, as well as the framework for collaboration and coordination, was developed in mutual agreement between the professions.

**Table 1:** Current clinical practice

	Action	Content
Before examination	Triage	The referral from the patient’s general practitioner is reviewed by an orthopaedic surgeon (OS) from The Shoulder Clinic. Depending on expected treatment plan, patients are allocated to one of three groups: 1) High probability of surgery or high complexity ➔ examination by an OS. 2) High probability of non-surgical treatment ➔ examination by an extended scope physiotherapist (ESP). 3) The remaining patients are distributed between ESPs or OSs based on first available time slot.
	Diagnostic imaging	X-ray of the glenohumeral and acromioclavicular joints are taken before examination of all patients.
	Patient reported information	Patients are asked to fill in a questionnaire including demographics and use of medications
On day of examination	Duration of examination	Time frames for the examinations are 20 min for OS and 30–40 min for ESP.
	Physical framework	ESP and OS perform the examinations in adjacent rooms.
	Diagnosis and treatment plan	The patient’s history is recorded based on ICF (International Classification of Functioning, Disability and Health). A clinical examination, including relevant diagnostic tests, is performed. In most cases an ultrasound imaging is also performed. Based on the findings, the patient is diagnosed. Based on a shared-decision process between the patient and the professional a treatment plan is decided on and initiated.
	Communication	ESP and OS consult if questions arise regarding diagnosis or treatment plan. It is mandatory for the ESP to consult an OS, if the treatment plan includes surgery, steroid injection or referral to advanced diagnostic imaging.
Throughout the year	Coordination and meetings	Complex patient cases and diagnostic imaging results are discussed on a weekly conference between ESPs and OSs. When needed, patient cases are also discussed outside the scheduled conferences. Four times a year, the entire team at The Shoulder Clinic meets to update, evaluate and optimize clinical pathways for patients.

**Table 2:** Extended scope physiotherapist specialist education program

	Action	Content
Time frame	Overall duration	1–2 years
	Duration in hrs.	602–818 h
Modules	Theoretical	• Clinical examination and diagnostic tests
		• Common differential diagnoses
		• Types of and indications for surgery
		• Evaluation of x-rays
		• Education in ultrasound imaging
		• Reporting in medical records
	Practical	• Sit-in during examination program. 4 days with an OS and 2 days with an ESP
		• Observation of 6–7 different kinds of surgery at the operating room
		• One year of practical training examining at least 400 patients in the shoulder clinic
Graduation	Completion of modules	Documented completion of the theoretical and practical courses
	Written exam	A written exam has to be passed
	Individual evaluation	The orthopaedic surgeon in charge of the education program evaluates the skills of the physiotherapist. In order to pass, the physiotherapist must be considered capable of: • Diagnosing common disorders of the shoulder and elbow using clinical examination, ultrasound imaging and evaluation of x-rays
		• Identifying common differential diagnoses
		• Initiating relevant and correct imaging
		• Initiating relevant and correct physiotherapy
		• Deciding whether consulting an orthopaedic surgeon is appropriate
		• Writing a correct medical report
		• Performing a postoperative follow-up on patients with an uncomplicated clinical pathway

### Design

This study was conducted at The Shoulder Clinic using a sequential mixed method design [31, 32] with emphasis on the quantitative component (i.e. dominant status) [33]. The study was conducted in two phases comprising two separate study designs. Both quantitative and qualitative data were collected and analyzed separately before being mixed at the stage of interpretation.

#### Study 1

In phase one (November 2017 to April 2018) an inter-rater agreement study with 69 participants was conducted (Aim 1a and 1b).

#### Study 2

In phase two (April 2018), a qualitative study with two focus group interviews was conducted (Aim 2).

### Participants

#### Study 1

Patients were recruited from The Shoulder Clinic. Inclusion criteria were: Referred with a shoulder disorder, considered equally appropriate to be examined by an OS or an ESP (see Table 1, triage: category 3), above 18 years and able to read and understand Danish.

Inclusion procedure: In random order (lottery draw), newly referred eligible patients were contacted by phone and invited to participate. If a patient was not reachable or declined to receive information about the study, another randomly chosen patient was contacted. After verbal information, if the patients considered participating, they received written information and were scheduled for examination according to study protocol. Written consent to participate was given on the day of examination. This procedure was replicated until the estimated sample size of at least 65 participants was reached.

#### Study 2

All ESPs and OSs working in The Shoulder Clinic in the inclusion period were invited and agreed to participate (three ESPs (CLL, SMRV, and SJD) and four OSs (including TMK)). All of them had more than 3 years of experience examining patients in this particular shoulder clinic. Three of the OSs were consultants and one was a specialty registrar.

### Data collection

#### Study 1

The examinations of the included patients were performed by the three ESPs and four OSs working at The Shoulder Clinic (see participants in Study 2). The patients were examined twice on the same day by both an ESP and an OS. In random order, they independently and blinded to each other performed the examinations. In a pre-developed registration chart (See Additional file 1), each professional registered diagnosis and treatment plan. The chart was developed by MLK and MNM in collaboration with both physiotherapists and orthopaedic surgeons from The Shoulder Clinic. To reduce risk of misclassification, ICD-10 codes (International Classification of Diseases and Related Health Problems – 10th Version) for each category were noted beneath the name of the diagnosis. Furthermore, the chart was pilot tested by the ESPs and OSs to ensure that all diagnoses used in clinical practice were covered and to clarify potential uncertainties.

To ensure that the examinations were performed fully independently, patients were instructed not to talk to the second assessor about, what was said and done during the first examination. Furthermore, the ESPs and OSs were explicitly instructed not to provide the patients with a diagnosis during the independent assessments.

##### Diagnosis

Nine categories were predefined: Subacromial impingement, rotator cuff injury, glenohumeral instability, glenohumeral osteoarthritis, adhesive capsulitis, scapula instability, fracture sequelae, acromioclavicular joint disorder, non-related shoulder diagnoses. Each professional registered one primary diagnosis and as many secondary diagnoses as considered relevant.

##### Treatment plan

Five categories were predefined: physiotherapy, steroid injection, diagnostic imaging, surgery, no intervention. Each professional registered as many categories of treatment as considered relevant.

Furthermore, each professional registered whether there was a need for follow-up at the shoulder clinic and/or a need for inter-professional consultation (indicating, if the professional in usual conditions would have consulted the other profession before deciding diagnoses and treatment plan). Also, the professional, who performed the first examination collected patient demographics regarding patient’s gender, age and employment status (employed, unemployed, retired, sick leave).

**Fig. 1:**
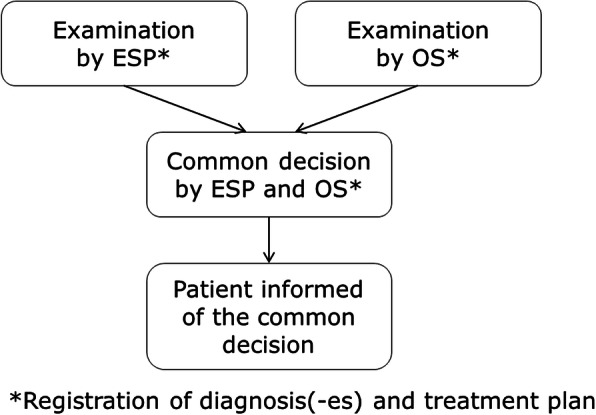
Procedure of examinations. ESP: Extended scope physiotherapist; OS: Orthopaedic surgeon

After each examination, the registration chart was collected by a research assistant and the professionals did not communicate between the examinations. After the two independent examinations, the professionals discussed their results and achieved a common decision, which was registered and passed on to the research assistant. Subsequently, the patient was informed of the common decision of diagnosis and suggested treatment plan. The patient was not informed of the individual results of the two examinations (Fig. 1).

#### Study 2

**Fig. 2:**
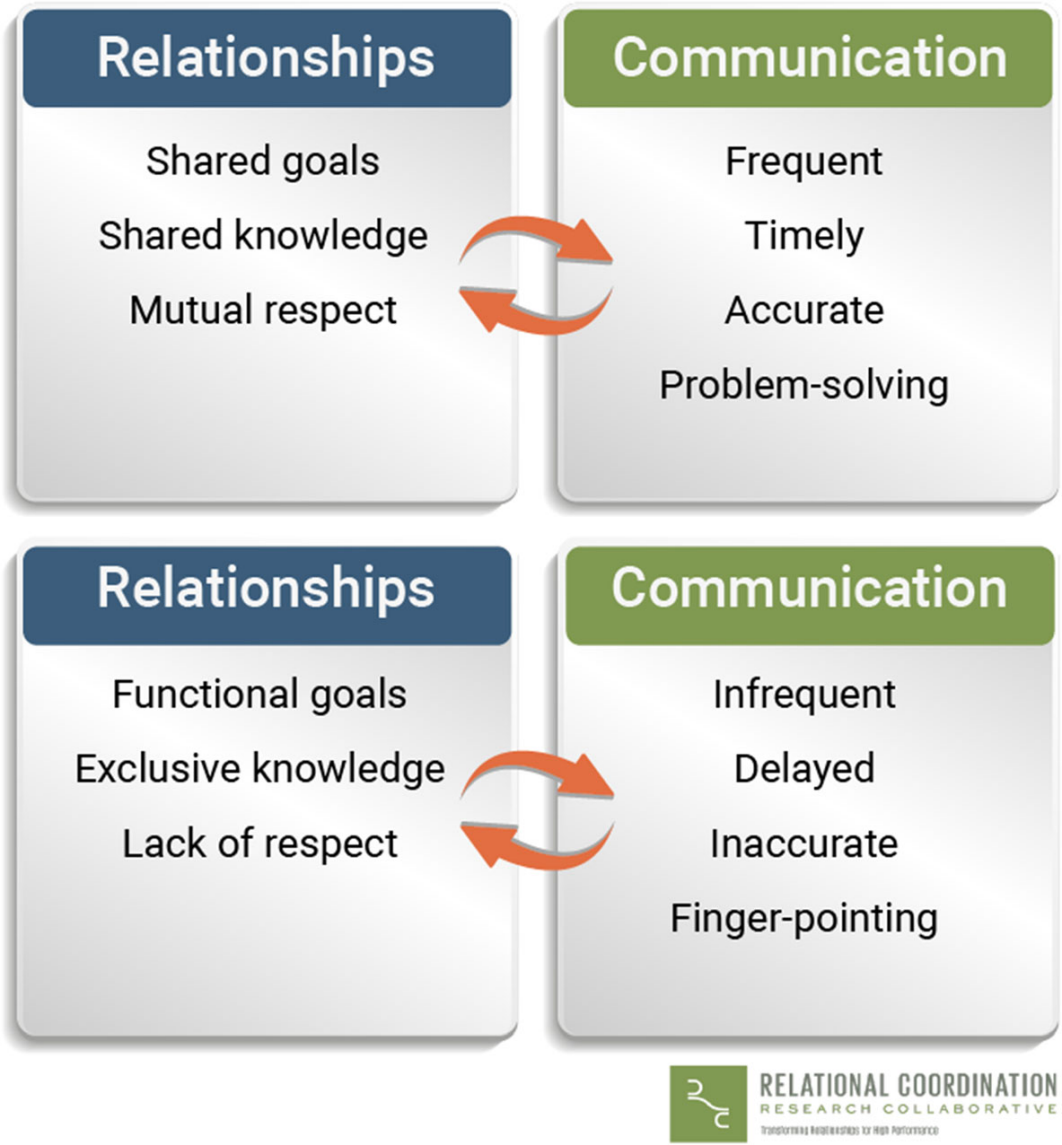
Relational coordination. The figure is used by permission of Relational Coordination Research Collaborative, Brandeis University [36]

The concept of relational coordination [34] was chosen as a theoretical framework for investigating the collaboration between ESPs and OSs sharing the task of examining patients with shoulder disorders. Relational coordination was found suitable, as it is defined as “a mutually reinforcing process of communicating and relating for the purpose of task integration” [35, 36] and focus on coordination of work with shared goals, shared knowledge and mutual respect between professionals [34, 37]. Furthermore, the concept has been used in several studies investigating quality and efficiency in the health care system [37, 38], and a high level of relational coordination has been shown to be positively associated with quality of patient care [26, 27]. Relational coordination consists of seven dimensions in total – four related to communication (frequent, timely, accurate and problem-solving) and three related to relationship (mutual respect, shared goals, shared knowledge). Figure 2 illustrates the dimensions of relational coordination and how they influence one another for better or for worse [36].

Two separate focus group interviews were conducted (by MLK) with OSs and ESPs, respectively to gain knowledge about their interpretations, experiences and opinions on interprofessional collaboration [39]. Separate interviews were chosen to increase the probability of participants speaking freely without being limited by neither a potential professional hierarchy (power relation) nor personal relations. A semi-structured interview guide was used, comprising questions based on the themes and dimensions of relational coordination [34] and inspired by the questions used in a previous Danish study [40]. Questions regarding overall evaluation of the inter-professional collaboration, and the professionals’ views on what it takes to be able to share the task of diagnosing patients, were also included (see Interview guide, Additional file 2). The focus group interviews were audio-recorded and subsequently verbatim transcribed.

The interviewer (MLK) was a master student with a background in public health, did not have a health professional education and was not employed at the hospital. MLK was introduced to the clinicians, the hospital procedures and terminology used in the health care system by MNM, who had been employed at Silkeborg Regional Hospital for a decade, and was well known by both researchers and clinicians at the Shoulder Clinic.

### Statistics and data analysis

#### Study 1

##### Sample size

Sample size was determined based on a nomogram from Hong et al. [41]. The following clinician-based estimates were used: Expected distribution of treatment plan (no intervention (expected 10%), physiotherapy (expected 30%) and a merged category comprising surgery/steroid injection/diagnostic imaging (expected 60%)), an expected simple proportion of agreement on 75% and a difference between proportions of agreement on 15%. Based on these parameters, and using α < 0.05 and β = 0.80, a sample size of 65 was required.

##### Descriptive statistics

Demographics and distribution of diagnoses and treatment plan are presented using descriptive statistics. Categorical variables are presented in numbers and percentages. Data on age was non-normally distributed and therefore reported in median and range.

##### Analyses of agreement

Comparisons were made both between ESPs and OSs but also between each profession’s decision and the common decision. Estimates of inter-rater agreement between ESPs and OSs are reported in numbers and proportions with 95% confidence interval (CI). When possible, the estimates are supplemented with Kappa (κ), prevalence-adjusted and bias-adjusted Kappa (PABAK), prevalence index and bias index [42, 43]. Likewise, estimates of agreement between the professions and their common decision are reported in numbers, and proportions with CI. Furthermore, in lack of a gold standard and assuming the common decision to be the most appropriate decision, risk difference between ESPs and OSs with CI and corresponding *p*-value is estimated.

Predefined estimates of diagnostic agreement:

1. Agreement on the primary diagnosis 2) Agreement on combination of diagnoses (defined as ESP and OS having registered the same diagnoses but with different rankings of primary/secondary) and 3) Partial diagnostic agreement, which was considered present if the primary diagnosis registered by one of the professionals was also registered as either primary or secondary diagnosis by the other.

Predefined estimates of agreement on treatment plan:

1. Agreement using three treatment categories (primary analysis). The three categories were “no intervention”, “physiotherapy” and “possibly invasive”. The latter consisted of the categories “surgery”, “diagnostic imaging” and “steroid injection” merged into one. In cases where both physiotherapy and a possibly invasive treatment were registered, the treatment plan was categorized as possibly invasive.
2. Agreement on each of the five treatment recommendations.

As a supplement to the primary analysis and the inter-rater agreement on surgery, in cases of disagreement, we described, whether the professionals had registered a need for inter-professional discussion or a new appointment in the shoulder clinic. This was chosen, assuming that these actions could optimize a presumable inadequate choice of treatment, thus being of clinical relevance.

Explorative estimates of agreement on treatment plan:

3) Total agreement on treatment plan defined as full concordance between categories chosen
4) Partial agreement defined as the professions agreeing on one or more recommendations on treatment.

Data entry was performed in Epidata 3.1 and data analysis in SPSS Statistics 25 and Stata 14.

**Changes from preregistered analyses in Clinical Trial**

In the analysis comparing the professions agreement with the common decision, a McNemar’s test was planned. However, based on statistical evaluation, risk difference with CI was considered to be of greater clinical relevance. Furthermore, the explorative analyses of agreement on combination of treatment plan were not preregistered in Clinical Trial, but were performed because in the primary analysis of agreement 93% of all cases were placed in one of three categories making this evaluation of agreement less meaningful. Also, a supplementary analysis of agreement on the combination of diagnoses was performed, as we considered it to be reflective of clinical practice.

A partial cost analysis was planned, but data available for the completion of the suggested treatment was imprecise and non-validated, and the costs per participant would cover different lengths of treatment pathway. Therefore, we found it not relevant to conduct as planned.

#### Study 2

A six phases thematic data analysis was performed inspired by Braun and Clark [44]. After familiarization with data, data was systematically elaborated to generate initial codes, which subsequently were categorized thematically in one of the seven themes (the dimensions of relational coordination). In an iterative process, the codes’ placements in each theme were reviewed in order to investigate, whether the predefined theme was fully representative and covered the content of the codes. Furthermore, the entire data material was once again scrutinized to identify details not covered. Themes and sub-themes were formed in a process where codes and data were examined in a back- and forth process. This was repeated until the themes identified were found representative of the inter-professional collaboration.

The process above, was performed by MLK in collaboration with her supervisor. Afterwards, CBR and MNM independently scrutinized the original data to validate and together with MLK adjust the identified themes. To further validate the findings, the results were presented to and considered accurate by the original participants.

## Results

### Study 1

**Fig. 3:**
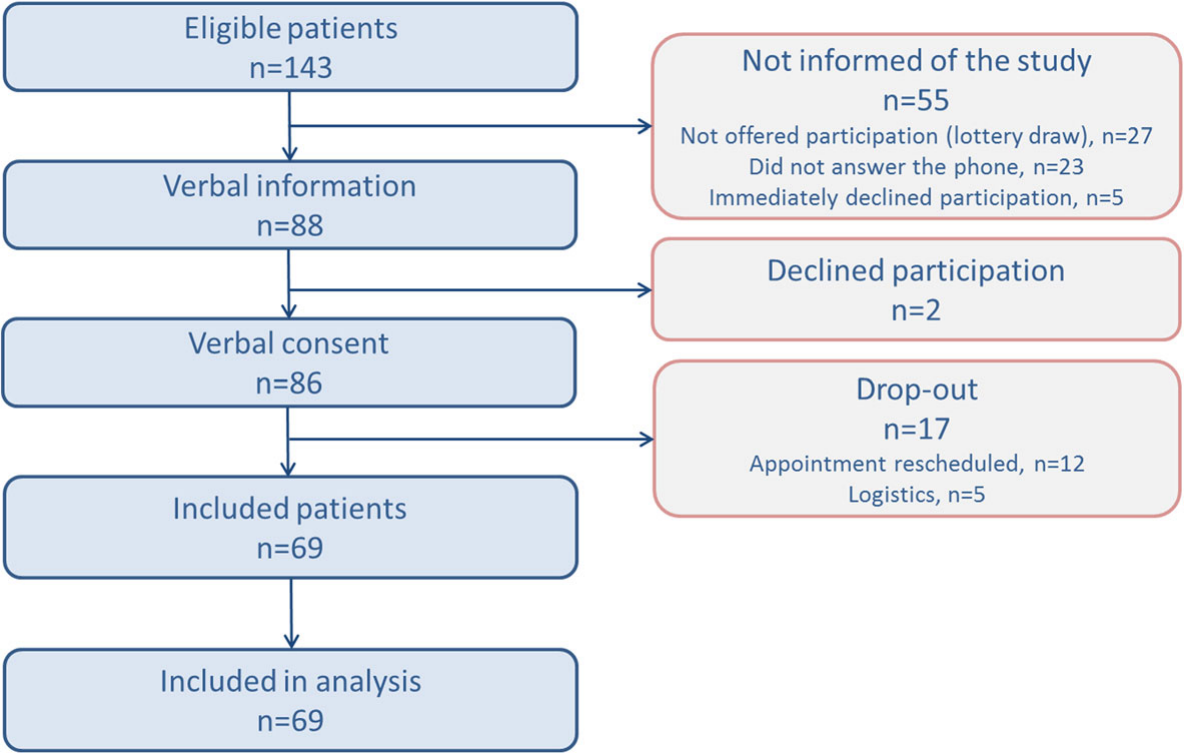
Participant flow

Sixty-nine patients were included in the study. All were included in the analysis (Fig. 3).

Patient demographics and distribution of diagnoses (based on the common decisions) are presented in Table 3. The most frequent primary diagnosis was subacromial impingement, which along with rotator cuff injury and adhesive capsulitis represented more than 82% of the primary diagnoses. The professionals registered maximum three and most frequently (55%) one secondary diagnosis. The two most frequently applied secondary diagnoses were subacromial impingement and acromioclavicular joint disorder. Details are presented in Supplemental Table 1, Additional file 3.

**Table 3:** Patient demographics and primary diagnoses (*n* = 69)

	Number (%) - unless otherwise stated
Gender	
Male	38 (55)
Female	31 (45)
Age, years	
Median [range]	54 [47–64]
Employment status (*n* = 63)	
Employed	31 (49)
Unemployed	3 (5)
Sick leave	7 (11)
Retired	22 (35)
Primary diagnoses	
Subacromial impingement	23 (33)
Rotator cuff lesion	11 (16)
Glenohumeral instability	1 (1)
Glenohumeral osteoarthritis	1 (1)
Adhesive capsulitis	12 (17)
Scapula instability	6 (9)
Fracture sequelae	0
Acromioclavicular joint disorder	13 (19)
Non-shoulder related diagnosis	2 (3)

Distribution of suggested treatment, need for follow-up and need for inter-professional consultation is presented in Table 4. In about half of the examinations the health professionals suggested two treatments, with steroid injection and physiotherapy being the most frequent combination. Based on the common decision, surgery was suggested in 3 (4%) of the cases and ‘No intervention’ was not registered in any cases. Also based on the common decision, the diagnostic imaging suggested was magnetic resonance imaging (MRI) in 17 cases, MRI + x-ray in one case) and magnetic resonance arthrogram (MR-arthrogram) in two cases.

**Table 4:** Distribution of suggested treatment, need for follow-up and need for inter-professional consultation

	ESPs Number (%)	OSs Number (%)	CDs Number (%)
**Suggested treatment**^**a**^			
Possibly invasive	65 (96)	63 (91)	64 (93)
Steroid injection	59 (87)	52 (75)	53 (77)
Diagnostic imaging	20 (29)	23 (33)	20 (29)
Surgery	4 (6)	5 (7)	3 (4)
Physiotherapy	41 (60)	39 (57)	38 (55)
No intervention	0 (0)	0 (0)	0 (0)
**New appointment**^**b**^			
Yes	65 (94)	61 (90)	62 (90)
**Inter-professional consultation**^**c**^			
Yes	49 (74)	4 (7)	NA
**Number of suggested treatments**^**a**^			
1	21 (31)	26 (38)	29 (42)
2	39 (57)	36 (52)	35 (51)
3	7 (10)	7 (10)	5 (7)
4	1 (2)	0 (0)	0 (0)

*ESP* Extended Scope Physiotherapist, *OS* Orthopaedic surgeon, *CD* Common decision

^a^ESP: *n* = 68, OS: *n* = 69, CD: *n* = 69

^b^ ESP: *n* = 69, OS: *n* = 68, CD: *n* = 69

^c^ESP: *n* = 66, OS: *n* = 59

#### Diagnostic agreement

ESPs and OSs agreed on the primary diagnosis in 62% (95% CI: [50.2; 73.3]) of the cases (κ = 0.51, PABAK = 0.56). In further 12 cases the professions agreed on the combination of diagnoses, but had a different ranking of primary/secondary, leading to a diagnostic agreement on the combination of diagnoses of 79% (95% CI: [70; 89]). Partial diagnostic agreement between ESPs and OSs was 96% (95% CI: [91; 101]).

When comparing each profession’s choices of diagnoses with the common decision, the OSs had a statistically significant higher agreement on the primary diagnosis compared with ESPs (Table 5).

**Table 5:** Agreement on diagnoses between ESPs, OSs and common decision (*n* = 69, unless stated otherwise)

	Agreement Number (%, [95% CI])		Risk difference^a^ Percent point [95% CI] *(p*-value)
	ESP	OS	
Agreement on primary diagnosis	47 (68, [57; 79])	62^b^ (91, [84; 98])	−23 [−36; − 10] (< 0.01)
Agreement on combination of diagnoses	60 (87, [77; 94])	65 (94, [86; 98])	−7 [−17; 2] (0.14)
Partial agreement	68 (99, [96; 101])	68 (100, [NA])	−2 [− 4; 1] (0.31)

*ESP* Extended Scope Physiotherapist, *OS* Orthopaedic surgeon

^a^Difference in percent point between ESPs and OSs with ESPs as reference (ESP-OS)

^b^ *N* = 68

#### Inter-rater agreement on treatment plan

##### Primary analysis

Agreement on treatment plan (three categories) between ESPs and OSs was 88% (95% CI: [81–96%]). Kappa was −0.06 and PABAK 0.76 (Details are presented in Supplemental Table 2, Additional file 3). The eight cases of disagreement concerned whether physiotherapy was suggested in combination with steroid injections (seven cases) and diagnostic examination (one case) or as the sole treatment. In all but one case of disagreement ESPs registered a need for inter-professional discussion or a new appointment in the shoulder clinic.

Across the five treatment categories, agreement between ESPs and OSs varied from 68% (physiotherapy) to 100% (no intervention) (Table 6). Disagreement on surgery was found in five cases and of those, surgery was registered as the common decision in one case (surgery registered by OS). In all five cases of disagreement, ESP registered both a need for inter-professional discussion and a new appointment in the shoulder clinic.

**Table 6:** Agreement between ESPs and OSs on treatment (*n* = 68)

Treatment category	Agreement Number (%, [95% CI])	Agreement Kappa	Agreement PABAK (PI; BI)
Physiotherapy	46 (68, [57; 79])	κ = 0.33	0.35 (0.18; 0.03)
Steroid injection	50 (74, [63; 84])	κ = 0.16	0.47 (0.62; 0.12)
Diagnostic imaging	55 (81, [72; 90])	κ = 0.56	0.62 (−0.37; − 0.04)
Surgery	63 (93, [86; 99])	κ = 0.41	0.85 (0.87; 0.01)
No intervention	68 (100, [NA])	NA	NA

Abbreviations: *CI* Confidence interval, *PABAK* Prevalence-adjusted and bias-adjusted kappa, *PI* Prevalence index, *BI* Bias index

##### Explorative analyses

Of 16 possible combinations of treatment categories, 13 were used. Total agreement between ESPs and OSs on the combined treatment plan was 43% (95% CI: [31; 54]) (κ = 0.27, PABAK = 0.38), while partial agreement was 96% (95% CI: [91; 100]).

#### Agreement on treatment between each profession and the common decision

Comparing each profession’s choice of treatment with the common decision, OSs had a higher level of agreement compared to ESPs. The difference was significant with respect to treatment (three categories), diagnostic imaging, and total agreement on combined treatment plan (Table 7).

**Table 7:** Agreement on treatment between ESPs, OSs and common decision

	Agreement Number (Percent, [95% CI])		Risk difference^a^ Percent point [95% CI] *(p*-value)
	ESP (*n* = 68)	OS (*n* = 69)	
Total treatment agreement (3 categories)	60 (88, [81; 96])	67 (97, [93; 101])	−9 [−17.5; − 0.3] (0.04)
Physiotherapy	55 (81, [72; 90])	60 (87, [79; 95])	−6 [−18; 6] (0.33)
Steroid injection	55 (81, [72; 90])	62 (90, [83; 97])	−9.0 [− 21; 3] (0.13)
Diagnostic imaging	58 (85, [77; 94])	66 (96, [91; 100])	−10 [− 20; − 1] (0.04)
Surgery	65 (96, [91; 101])	67 (97, [93; 101])	−2 [− 8; 5] (0.64)
No intervention	68 (100, [NA])	69 (100, [NA])	NA
Total treatment agreement (15 categories)	38 (56, [44; 68])	52 (75, [65; 86])	−20 [−35; −4] (0.01)
Partial treatment agreement (15 categories)	67 (99, [96; 101])	68 (99, [96; 101])	−0 [−4: 4] (0.99)

*ESP* Extended Scope Physiotherapist, *OS* Orthopaedic surgeon

^a^Difference in percent point between ESPs and OSs with ESPs as reference (ESP-OS)

### Study 2

Data analysis showed that all seven dimensions of relational coordination were highly and positively present. In the thematic analysis we found three themes to be especially important when ESPs and OSs were collaborating in the shared task of diagnosing patients: Close communication, equal and respectful relationship and professional skills.

#### Close communication – enhanced by the setting

Both ESPs and OSs described, that the opportunity of ad hoc inter-professional communication and consultation during the day had a positive impact on their work.

*"I feel it is unique that we just can go next door without the sense of disturbing and solve challenges in a swift manner" (OS 2)*

The respondents reported, that this access increased their ability to quickly clarify questions and doubts and make final decisions on i.e. best possible treatment choice for the patients. This was also mentioned to be of importance for timely initiated service to the patients, as patients may be spared a second visit at the clinic. According to the OSs, it was considered crucial, that ESPs and OSs performed the examination of patients in rooms located close to each other in order to enable the professionals to consult each other ad hoc during the day work. Both ESPs and OSs described their inter-professional communication to be accurate. The frequency of communication during the day varied and was based on a mutual understanding of indications for consulting each other. The ESPs consulted the OSs more often than the other way around. The OSs were confident, that ESPs consulted them when needed and in a timely manner.

#### Equal and respectful relationship – playing the same tune

ESPs and OSs described, that when consulting the other part, they were met with a helpful and receptive attitude and had a positive dialogue. Also, they emphasized the importance of feeling comfortable with each other in order to seek advice in a timely manner and whenever needed.

ESPs mentioned, that they were aware they did not have the same level of education and experience as the OSs, but still both ESPs and OSs explicitly expressed, that a feeling of equality was of importance for the collaboration. This mutual feeling of equality was based on the fact, that they had both some competences in common but also some competences specific to each profession, making them able to help each other both ways. Both groups mentioned, that the fact, that the ESPs were also used for rehabilitation of shoulder patients at the hospital, contributed to this.

*"Of course they (ESPs) have their own professional approach; however, it also challenges me and has changed my views on many things. And that is exactly what we should gain from collaboration; exchange of ideas and opinions in order to make improvements" (OS 3)*

*"We are fully aware not to be at their (OSs) level, you know, when it comes to education and experience. [ … ] As for conservative treatment – well, here we have certain competences – while obviously [surgery] is totally their subject. So, we all contribute with our different strengths, and this also promotes respect" (ESP 3)*

In both groups, good collaboration was also described as an asset based on personal relations. It was considered important to have a positive attitude to helping each other and working together in this field. They had a shared goal of providing patients with the best possible service.

*“If there is a disagreement, then it is dealt with in a pleasant and respectful manner. We are never scolded or corrected in a degrading manner that makes us feel stupid. The surgeons are listening and ask many clarifying questions. This gives at sense of respect and they show a genuine interest in helping" (ESP 2)*

*"We are playing the same tune (OS 1). Yes, that's what it feels like (OS 2). There needs to be a common understanding between physiotherapists and surgeons (OS 1)"*

#### Professional skills – importance of the education program

ESPs and OSs reported mutual respect, which was promoted by their professional skills.

*"They have been through a proper educational program, not some random course. We have been very determined about the education. We did not want to work with physiotherapists who just had good intentions and no proper knowledge. That is why we keep having weekly group conferences where we can discuss MRI and X-ray imaging, complex cases and have general discussions" (OS 2)*

Professional skills were shared through the ESPs’ education program, consultations/dialogue and conferences, leading to increased skills, shared knowledge and a mutual understanding of how to supplement each other. Both ESPs and OSs highlighted the ESP education program as beneficial. ESPs described, that it made a major contribution to their ability to work more independently and the OSs mentioned it as an important part of the reason for ESPs’ professional skills to diagnose patients.

## Discussion

### Summary of results

In the present study the diagnostic agreement between ESPs and OSs was 62% for primary diagnosis, 79% for the same combination of diagnoses and 96% for partial agreement on the diagnoses (the primary diagnosis registered by one of the professionals was also registered as either primary or secondary diagnosis by the other). The OSs had a significant higher agreement with the common decision on the common decision than the ESPs. In the predefined primary analysis, ESPs and OSs agreed on the treatment plan being either possibly invasive, physiotherapy or no intervention in 88% of the cases. Due to a skewed distribution of data (93% allocated to possibly invasive treatment) this result was considered less meaningful than expected. Across treatment categories the agreement between OSs and ESPs varied between 68 and 100%, with OSs having a significant higher agreement with the common decision on the need for diagnostic imaging. In 43% of the cases, ESPs and OSs agreed on the combined treatment plan and in 96% of the cases they partially agreed.

In the evaluation of collaboration between ESPs and OSs, data showed positive statements regarding all seven dimensions of relational coordination. We found close communication, equal and respectful relationship and professional skills to be especially important.

In the following, the quantitative results related to aim 1a (agreement on diagnoses) and 1b (agreement on treatment plan) are discussed integrating some of the qualitative findings in the interpretation of the results.

### Agreement on diagnoses

We have only been able to identify two studies, which have reported inter-observer agreement between ESPs and OSs when diagnosing patients with shoulder disorders, thus being eligible for comparison with our results [15, 23]. Among seven categories, Razmjou et al. [15] reported a diagnostic agreement between ESPs and OSs on each category varying between 84 and 98%. In their study, they used different diagnostic categories compared to our study and allowed for selecting more than one diagnosis without differentiating between primary/secondary [15]. This means, that our estimates cannot be directly compared, and the 62% agreement on primary diagnosis was expectedly lower than Razmjou’s estimate of agreement in each category. In a retrospective audit on selected shoulder patients, a fully comparable diagnostic agreement of 65% and partial agreement on further 31% were reported [23]. Adding it up, it makes their result similar to the 96% of cases with partial diagnostic agreement in our study.

Furthermore, we found 79% agreement on the combination on diagnoses. Considering the fact, that in our setting, it is not clinical practice to distinguish between primary and secondary diagnoses, this estimate is probably the most clinically relevant. To our knowledge, no other studies have reported a similar estimate making comparison with other studies challenging. It is, though, comparable to general results on diagnostic agreement, when looking at studies investigating different musculoskeletal conditions [12].

To determine if our results reflect acceptable quality several factors should be considered. A 100% agreement cannot be expected, as diagnosing patients with shoulder disorders is a complex procedure, which takes comprehensive clinical reasoning skills in addition to performing specific tests to make a diagnosis. Some degree of subjectivity cannot be eliminated, and intra-professional agreement among OSs or ESPs is not expected to be perfect either. We haven’t been able to identify studies conducted in a setting similar to ours, that verify this, but the assumption is supported by an older study evaluating the agreement between three rheumatologists diagnosing 44 patients with shoulder disorders [45]. A 46% complete agreement was found, but it should be noted, that it will always be more difficult to get full agreement between three persons than two. In the same study, further 18 patients were examined by all three together where after they discussed and agreed on symptoms and signs, before individually writing the diagnosis and recommended treatment. With this procedure 78% agreement were reported [45], which underlines the impact of clinical reasoning.

It should be considered also, that there is no gold standard available for comparison. In cases of disagreement, we cannot determine the correct choice. Even in cases with agreement, a previous study has reported the diagnosis could still be incorrect [22], and although this was an example based on a knee disorder, it is reasonable to expect, this could also be the case among patients with shoulder disorders.

In this study, we used the common decision as the assumed most correct diagnosis(−es). Comparing each of the professions with the common decision, OSs had a significant higher agreement than the ESPs. This could be due to better professional skills, supported by the fact, that the OSs in this study were highly experienced consultants or specialty registrar. This is to some degree supported by the findings in our qualitative study. Although, an equal and respectful relationship were revealed, still, the physiotherapist acknowledged, that the OSs had more experience and another level of education. This consideration could also be part of the explanation why the ESPs registered need for inter-professional consultation in 74% of the cases, compared with 7% registered by the OSs. The difference in proportions was not surprising, since the patient was referred for an orthopaedic specialist evaluation and the ESPs practice under the responsibility of the OSs. It was also expressed in the qualitative study, that ESPs consulted the OSs more often than the other way around. We did not collect data on reasons why the ESPs needed inter-professional consultation. However, during interviews a mutual understanding of indications for inter-professional consultation was expressed, hence, the OSs in our setting presumably agrees, that consultations were relevant.

Looking at further findings from the qualitative study, the ESPs’ high proportion of 74% could also be interpreted, as the ESPs feeling so comfortable with the OSs, that they sought advice whenever needed. Furthermore, even though the OSs only registered need for inter-professional consultation in 7% of the cases, the results reflect the finding of ESPs and OSs having a mutual understanding of having some competences in common but also having some competences specific to each profession.

Returning to the fact, that OSs had a higher level of agreement with the common decision compared to the ESPs, it should also be considered an explanation, that the task is traditionally managed by the OSs and the historical hierarchy of OSs being placed higher than physiotherapists.

### Agreement on treatment

We predefined the primary analysis as a comparison of the agreement on treatment plan (three categories). This choice was clinically based, because we considered it highly important to decide if a possibly invasive treatment was a relevant option for the patient. However, the data distribution between categories (93% in the category “possibly invasive”) made it less meaningful to use as an indicator of quality. Although, we showed an 88% agreement between ESPs and OSs, based on Kappa, this was not higher, than what could have been achieved by chance. However, due to the skewed data distribution, this was expected, and when calculating PABAK, it was 0.76. Thus, we still consider the agreement on 88% to be satisfactory. In seven of the eight cases where ESPs and OSs didn’t agree, it concerned whether the patient should be offered a steroid injection in adjunction to physiotherapy or not. This was not surprisingly the issue, as these two treatment types are often prescribed simultaneously, in patients with shoulder disorders caused by subacromial impingement [46]. In one case only, the physiotherapist had not registered either need for inter-professional discussion or a follow-up visit, thus in usual clinical practice, the disagreement would have been solved this way in all cases but one. This underpin the findings from Study 2, highlighting the importance of close communication, enhanced by using a setting allowing for the opportunity to do immediate communication per need.

The inter-professional agreement across the five treatment categories varied between 68 and 100% with a 93% agreement on indication for surgery. In this study, only three patients were immediate candidates for surgery, and this low proportion inevitable leads to a reduced kappa-value (0.41, indicating moderate agreement). The corresponding PABAK was 0.86, which support, that the low proportion of candidates for surgery is the main reason why our kappa-value is lower than the 0.75 (PABAK = 0.76) reported in the study of Razmjou et al. [15], in which, around half of the patients required surgical consultation. Our proportional agreement on surgery is nonetheless considered high and similar to the 88% reported by Razmjou et al. [15] and the 86% of cases in the study of Oakes et al. [23], where ESPs correctly predicted which patients would undergo surgery.

In our study, 20 participants (29%) were referred to diagnostic imaging (MRI or MR-arthrogram) and the overall inter-rater agreement was 81% (κ = 0.57, PABAK = 0.62). In comparison, Razmjou et al. [15] reported agreement on each category separately and found 91% agreement on MRI and 97% on MR-arthrogram. However, due to a small proportion of their population referred to these investigations, they reported lower kappa-values, 0.27 (PABAK = 0.86) and 0.38 (PABAK = 0.94) respectively. The differences in analyses and proportion of patients referred to MRI should be taken into consideration when comparing our results, but all together, we find our results to be comparable.

In the explorative analyses, the combined treatment plan was found to be exactly the same for the ESPs and the OSs in 43% of the cases and partially the same (at least one category in common) in 96% of the cases. To our knowledge, there are no other studies reporting similar estimates for comparison. To evaluate, whether these estimates indicate satisfactory quality, different factors should be taken into consideration. First, the total agreement would expectedly be quite low, as there were 16 different possible combinations available – and 13 were actually used. Second, the high partial agreement could be affected by the professionals’ option to choose as many categories as considered relevant, thus increasing the chance of reaching agreement on at least one treatment. However, this is not considered a great concern, as both professions chose no more than one or two treatments in about 90% of the cases.

In lack of a gold standard, we used the common decision as the assumed best choice. When comparing the treatment plans suggested by ESPs and OSs with the common decision, it is important to note, that there was no statistical difference between the professions on indication for surgery. ESPs differed from the common decision in three cases and OSs in one case. This exemplifies, that both professions could change their view based on inter-professional consultation, even when it comes to indication for surgery. This result supports the qualitative finding of an equal and respectful relationship, and their mutual trust in each other’s professional skills.

The absence of a statistical difference between the professions on indication for surgery is positive, when comparing with Razmjou et al., where the ESPs tended to recommend surgery more often than the OSs [15]. But, compared to the common decision, the OSs in our study had a significantly higher agreement than ESPs with respect to treatment (three categories), diagnostic imaging and total agreement on the combined treatment plan. Reasons for this difference are similar to those discussed for diagnostic agreement.

In summary, when comparing with other study results and considering factors challenging a perfect agreement, we believe that our results of both diagnostic and treatment agreement indicate a satisfactory level of quality when evaluating ESPs and OSs sharing the task of diagnosing patients with shoulder disorders. In the interpretation of the quantitative results, it is important to note, that close communication, equal and respectful relationship and professional skills, seems to contribute to being able to successfully share the task.

### Collaboration

The findings of our study indicate a high degree of relational coordination between ESPs and OSs highlighting close communication, equal and respectful relationship and professional skills. Based on the indication of a high relational coordination, the overall interpretation of the qualitative data was, that inter-professional collaboration was good. Our findings indicate, that our setting and the collaboration between ESPs and OSs, meets a great deal of the needs and recommendations mentioned in other studies [19, 30] as well as The World Confederation for Physical Therapy (WCPT) Policy Statement: Advanced Physical Therapy Practice [47]. In the study of Weatherley et al. it was described, that to provide patients with the best care, the referrals should be appropriate and the physiotherapist should receive support from the surgeon [19]. Our data shows, the support of the OSs is undoubtedly present, and we also found indications of a well-functioning referral. In our setting, based on the referral from the patient’s general practitioner, a selected group of patients are deemed equally eligible to be examined and diagnosed by an ESP or an OS (Table 1). A criterion for this is the patient not being a clear candidate of surgery. This is highly fulfilled in our study population, where three patients only, were immediately recommended surgery. Even though a higher proportion of surgery candidates are expected later in the process (e.g. after diagnostic imaging), the referral is considered highly adequate. In the study of Dawson and Ghazi, it is concluded, that ensuring a good relationship between ESPs and the medical team as well as providing adequate ongoing training and support, could minimize many of the difficulties encountered by the ESPs [30]. Furthermore, in the same study, one of the recommendations for the future were that ESP positions should be set up at the request of, or with full back-up from the orthopaedic team [30]. This description fits perfectly the way our setting evolved, and the findings in our study revealed, that the ESPs experienced full back-up from the OSs. Some of the other recommendations were: adequate time allowed for shadowing; basic training in requesting and reading X-rays, blood results and understand common pharmacology; and responsibilities and expectations defined at onset [30]. To a great extent, these recommendations are met by the ESP education program established at our hospital. The provision of appropriate education is also advocated in the World Confederation for Physical Therapy policy statement [47], thus underlining the importance of having adequate professional skills to manage the post of being an ESP.

To our knowledge, no previous studies have investigated the experience of ESPs in the light of professional quality. In this study, we found indications of both a high level of relational coordination between ESPs and OSs as well as a satisfactory level of agreement on diagnoses and treatment plan, thus an association may be present. This is in accordance with studies in other field of health care, showing a positive association between relational coordination and quality of patient care [26, 27]. It was beyond the scope of this study to investigate and describe how to best establish a new position for an ESP and how to achieve a good collaboration. However, we have thoroughly described our setting and how it evolved, as well as our education program (Table 2). Along with specific recommendations from other studies [30, 47], this description may be used as an inspiration.

### Study strengths and limitations

The strengths of the inter-rater agreement study are the examinations being performed in a randomized sequence, thus avoiding systematic bias from potential changes of patient history or reaction to tests based on the previous examination. Furthermore, the ESPs and OSs performed the examinations fully independent, blinded to each other’s findings and on the same day. Also, the patients were blinded to the diagnosis and treatment plan suggested by the ESP and OS, respectively, and to further avoid referral of information between examinations, participants were instructed not to talk to the second assessor about what was said and done at the first examination. Altogether, the risk of information bias is considered low and expectedly reduced compared to the previous studies investigating inter-rater agreement between ESPs and OSs examining patients with shoulder disorders [15, 23]. Finally, all patients included in the study were included in the analyses, thus no attrition bias is present. However, our results can only be generalized to the group of patients with shoulder disorders not being clear candidates of surgery, as this was part of the eligibility criteria. Still, in our setting, this group accounts for about 70% of all patients referred to The Shoulder Clinic, thus making it relevant for the majority of patients.

The registration chart for diagnosis and treatment were self-developed, thus not scientifically validated. This was chosen to reflect clinical practice and also because, due to our knowledge, there is no consensus on how to group diagnoses in the most appropriate manner. However, we minimized the risk of misclassification by using ICD-10 codes in each category and increased content validity by pilot-testing the chart to ensure that all diagnoses were covered. Based on this, we consider the risk of misclassification to be low.

In the qualitative study, we aimed to ensure trustworthiness as described by Miles, Huberman and Saldana [48]. Some of the study’s strengths and limitations of importance for trustworthiness are discussed below. Two separate focus-group interviews were performed to prevent bias (presumably in the positive direction) when describing the collaboration. We also consider it a strength, that the findings were confirmed by three researchers and considered accurate, when being presented to the original participants. Furthermore, as all ESPs and OSs at The Shoulder Clinic participated, data was collected across the full range of respondents and participants were fully representative for our setting. However, although being fully representative for our setting, the results can only be generalized to highly experienced and educated clinicians (both ESPs and OSs) in similar settings.

The use of relational coordination as theoretical framework and semi-structured interviews were chosen to evaluate dimensions of collaboration previously shown to be of importance for quality. However, this framework may not be sufficient to fully describe the inter-professional collaboration. First, it does not comprise power relations, which could also have an impact on the collaboration [49, 50]. Second, in our study, both the setting (including physical framework) and professional skills were contributors to the good collaboration, and neither of these factors is explicitly comprised in the seven dimensions of relational coordination. Thus, using semi-structured interviews primarily based on relational coordination implies the risk of missing some important areas. We tried to counter for this, by also asking the participants for overall factors necessary for a good collaboration, but still, the data produced cannot be interpreted as exhaustive.

A potential cognitive bias could be a positive attitude towards ESPs sharing the task of diagnosing patients with OSs, since several authors are employed at The Shoulder Clinic (TMK, CLL, SMRV, SJD and BEL) and others are Physical Therapists (MNM, LRM and JT). To counter for this, the main part of the analyses was performed by an author without a health professional education (MLK) and not employed in The Shoulder Clinic.

Finally, based on our results, we cannot evaluate overall quality of ESPs and OSs sharing the task of examining patients with shoulder disorders. We have investigated – and shown positive results on - some of the indicators of professional and organizational quality, but it would be important to know the outcome of treatment for patients, the cost-effectiveness and the patient-perceived quality as well to fully evaluate the quality. Presumably, ESPs and OSs sharing the task of diagnosing patients with shoulder disorders has the potential to reduce costs, optimize use of specialist consultant competences, and increase quality – especially by using inter-professional competences in patients with unclear clinical pictures. However, to establish scientific evidence of quality in this broad range of areas, further research – especially based on high quality randomized controlled trials - are needed.

## Conclusions

In the majority of cases, the ESP and OS agreed on the same or partly the same diagnosis and treatment plan. There were indications of a high degree of relational coordination between the professions, supporting good collaboration. This study supports that, when good collaboration enhanced by sufficient settings and professional skills, exists, ESPs and OSs can share the task of examining a selected group of patients with shoulder disorders in an orthopaedic clinic.

## Supplementary Information

**Additional file 1.** Registration charts. The file includes the registration charts used for both the individual examinations as well as registration of the common decision.

**Additional file 2.** Interview guide. The file presents the interview guide used in the focus group interviews.

**Additional file 3.** Supplemental results. The file includes two supplemental tables: Distribution and number of diagnoses registered, and Agreement between ESPs and OSs on suggested treatment (three categories).

## Data Availability

The datasets generated during the current study are not publicly available due to them containing information that could compromise research participant privacy/consent. The part of data, that can be anonymised, can be made available from the corresponding author upon reasonable request.

## Abbreviations

CI: Confidence Interval
ESP: Extended Scope Physiotherapist
ICD-10: International Classification of Diseases and Related Health Problems – 10th Version
ICF: International Classification of Functioning, Disability and Health
κ: Kappa
MSC: Musculoskeletal condition
OS: Orthopaedic surgeon
PABAK: Prevalence adjusted and bias adjusted kappa
