# Supplementary material for: Inter-professional agreement and collaboration between extended scope physiotherapists and orthopaedic surgeons in an orthopaedic outpatient shoulder clinic – a mixed methods study
Source: BMC Musculoskelet Disord. 2021 Jan 4;22:4. doi: 10.1186/s12891-020-03831-z (PMC7784386; doi:10.1186/s12891-020-03831-z)
Supplement: Supplementary file 1 — Additional file 1. Registration charts. The file includes the registration charts used for both the individual examinations as well as registration of the common decision. [file 12891_2020_3831_MOESM1_ESM.pdf]

Additional file 1, Registration charts

Registration of diagnoses and treatment

Do not discuss the case with other health professionals during the examination and do not communicate your results to the patient.

Participant number :_____________ Name:____

Date:__________________ Name of examiner:_______________________________________

**Diagnosis**<br>Select one primary diagnosis only.<br>If relevant, one or more secondary diagnoses may be selected.	Mark one -- and one only<br>**Primary diagnosis**	Mark one or more, if relevant<br>**Secondary diagnosis**
**Subacromial impingement** (including biceps tendinitis)<br>ICD-10 codes: DM 75.4, DM 75.3, DM 75.3A, DM 75.1, DM 75.2		
**Rotator cuff injury** (including biceps longum rupture)<br>ICD-10 codes: DS 46.0, DS 46.1B, DM 75.1C		
**Glenohumeral instability**<br>ICD-10 codes : DM 24.4, DM 24.4D, DS 46.1C, DS 43.0, DM 25.3, DM 35.7		
**Glenohumeral osteoarthritis**<br>ICD-10 codes : DM 19.8A, DM 19.0*		
**Adhesive capsulitis**<br>ICD-10 codes : DM 75.0		
**Scapula instability**<br>ICD-10 codes : DM 75.8, DM 75.9		
**Fracture sequelae**<br>ICD-10 codes : DT 92.1		
**Acromioclavicular joint disorder** (including sternoclavicular joint disorder)<br>ICD-10 codes : DM 19.8, DM 19.0*, DS 43.1, DS 43.2, DS 43.5, DS 43.6		
**Non-related shoulder diagnosis**<br>ICD-10 codes : among others, DM 54.2		

*DM19.0 can both be used when osteoarthritis is present at the glenohumeral joint as well as the acromioclavicular joint. Please be aware to mark the appropriate box.

**Treatment plan**<br>Mark one or more
**Referral to physiotherapy**<br>**Type:** Municipality: ☐ Further ESP assessment: ☐ Hospital: ☐ Private clinic: ☐
**Steroid injection**<br>Diagnostic purpose: ☐ Treatment: ☐
**Diagnostic imaging** (including referral to other paraclinical assessments or other specialists)<br>Referral to diagnostic imaging or other paraclinical assessments:<br>X-ray: ☐ CT-scan: ☐ MRI: ☐ MR arthrogram: ☐ ENG/EMG: ☐ Other: ☐<br>Referral to another specialist: ☐ Describe:____________________
**Surgery** ☐
**No intervention** (none of the above)<br>Patient is provided with: Recommendations (including use of pain medication): ☐<br>Recommendation to consult general practitioner: ☐ No recommendations: ☐

**Follow up and inter-professional consultation**
**Follow up** Yes: ☐ No: ☐ If yes, which professional should do the follow up. ________________
**Need for inter-professional consultation**[1]: Yes: ☐ No: ☐

[1] Mark this if you during normal conditions would have consulted the other profession before deciding diagnosis and treatment plan.

Additional file 1, Registration charts

Registration form – <u>common decision</u>

After the two independent examinations, ESP and OS discuss their results and make a common decision on diagnosis(es) and treatment plan. The health professional, who did the first examination of the patient, is responsible of filling in this registration form, of passing on the information to the patient and of writing the medical record of the patient.
The decision on actual initiated treatment is a common decision between examiner and patient, and potential deviations from the "common decision" should NOT be reported in the form beneath.

Participant number :_______ Name:____
Date:_________________ Name of professional filling in this form___________________________

Demographics	
**Gender** Female: ☐ Male: ☐	**Age**: ________ years
**Employment status**<br>Employed: ☐ Unemplyed: ☐ Pensioner/retired: ☐ Sick leave:: ☐ Other:_________________	

**Diagnosis**<br>Select one primary diagnosis only.<br>If relevant, one or more secondary diagnoses may be selected.	Mark one -- and one only<br>**Primary diagnosis**	Mark one or more, if relevant<br>**Secondary diagnosis**
**Subacromial impingement** (including biceps tendinitis)<br>ICD-10 codes: DM 75.4, DM 75.3, DM 75.3A, DM 75.1, DM 75.2		
**Rotator cuff injury** (including biceps longum rupture)<br>ICD-10 codes: DS 46.0, DS 46.1B, DM 75.1C		
**Glenohumeral instability**<br>ICD-10 codes : DM 24.4, DM 24.4D, DS 46.1C, DS 43.0, DM 25.3, DM 35.7		
**Glenohumeral osteoarthritis**<br>ICD-10 codes : DM 19.8A, DM 19.0		
**Adhesive capsulitis**<br>ICD-10 codes : DM 75.0		
**Scapula instability**<br>ICD-10 codes : DM 75.8, DM 75.9		
**Fracture sequelae**<br>ICD-10 codes : DT 92.1		
**Acromioclavicular joint disorder** (including sternoclavicular joint disorder)<br>ICD-10 codes : DM 19.8, DM 19.0, DS 43.1, DS 43.2, DS 43.5, DS 43.6		
**Non-related shoulder diagnosis**<br>ICD-10 codes : among others, DM 54.2		

**Treatment plan**<br>Mark one or more
**Referral to physiotherapy**<br>**Type:** Municipality: ☐ Further ESP assessment: ☐ Hospital: ☐ Private clinic: ☐
**Steroid injection**<br>Diagnostic purpose: ☐ Treatment: ☐
**Diagnostic imaging** (including referral to other paraclinical assessments or other specialists)<br>Referral to diagnostic imaging or other paraclinical assessments:<br>X-ray: ☐ CT-scan: ☐ MRI: ☐ MR arthrogram: ☐ ENG/EMG: ☐ Other: ☐<br>Referral to another specialist: ☐ Describe:____________________
**Surgery** ☐
**No intervention** (none of the above)<br>Patient is provided with: Recommendations (including use of pain medication): ☐<br>Recommendation to consult general practitioner: ☐ No recommendations: ☐

**Follow up**
**Follow up** Yes: ☐ No: ☐ If yes, which professional should do the follow up. ___________________
