# Supplementary material for: Inter-professional agreement and collaboration between extended scope physiotherapists and orthopaedic surgeons in an orthopaedic outpatient shoulder clinic – a mixed methods study
Source: BMC Musculoskelet Disord. 2021 Jan 4;22:4. doi: 10.1186/s12891-020-03831-z (PMC7784386; doi:10.1186/s12891-020-03831-z)
Supplement: Supplementary file 2 — Additional file 2. Interview guide. The file presents the interview guide used in the focus group interviews. [file 12891_2020_3831_MOESM2_ESM.pdf]

Additional file 2, Interview guide

Interview guide

Thank you for participating in this focus group interview about the collaboration between physiotherapists and orthopaedic surgeons when sharing the task of examining patients with shoulder disorders. We are going to talk about your way of communicating and your professional relationships. This will not be a typical interview with lots of questions from me, but instead, I will ask you some preliminary questions that you are going to answer and discuss. I am interested in all your experiences and perceptions on your collaboration, thus, no answers are right or wrong. You will all be anonymous when the results of this study are being presented. To help my memory, I would like to audio record the interview. Is that okay?

Research topic		Question
Opening topics		
Education and experience		One by one, please elaborate on you educational background and for how long you have evaluated patients with shoulder disorders.
Work		Please explain how you experience a normal work day in the shoulder clinic?
Relational coordination		
Dimensions of Relational coordination	*Questions previously used to measure relational coordination in a questionaire*[1]	
Frequency	How often do you speak or write to you colleagues regarding your work with patients?	On a normal work day, how often do you have contact with an orthopaedic surgeon/physiotherapist? Contact can include professional discussion of a specific patient case, general discussions about issues related to patients or just sharing experiences you had during a patient consultation.
Timeliness	Do you colleagues speak or write to you in a timely manner?	Do the orthopaedic surgeons/physiotherapists tell you the things you need to know at the proper time points?
Accuracy	Do your colleagues speak or write to you in a manner that you precisely understand?	Do the orthopaedic surgeons/physiotherapists communicate clearly and in a way that you easily understand? What do you do if the communication is unclear?
Shared goals	Do you and your colleagues share goals with regards to the patients?	What do the orthopaedic surgeons/physiotherapists need to do in order for you to perform you tasks? Do they do what's needed? Do they sometimes do things differently?
Problem solving	When work related problems occur, do you colleagues collaborate in solving the problem or try to blame others?	Exercise: Individually using a scale from 1-5 please rate to which degree the orthopaedic surgeons/physiotherapists assist you if difficulties/questions arise during a patient consultation. (1: not at all 2: rarely 3: partly 4: often, 5: always).<br>Why did you pick that number?<br>Please give examples that support you rating.
Mutual respect	Do your colleagues respect you role in working with patients?	Do you feel that the orthopaedic surgeons/physiotherapists respect you? Please give examples.<br>Do you respect the orthopaedic surgeons/physiotherapists?
Shared knowledge	How much do your colleagues know about your work?	Do you feel that the orthopaedic surgeons/physiotherapists are aware of you contribution to the shoulder examinations? Do they know what you do?<br>Do you know what the orthopaedic surgeons/physiotherapists do?

[1] Questions were used in the project (Danish) 'Relationel koordinering i hverdagsrehabilitering' (Albertsen, K. and I.-M. Wiegman, *Relationel Koordinering - om tværfagligt samarbejde og kommunikation.* Karnov Group Denmark A/S, 2014: p. 1 – 5)

Additional file 2, Interview guide

Evaluation of collaboration	Exercise: Finally, on a scale from 1-5, please rate to which degree you think that the shared task of shoulder examinations between physiotherapist and orthopaedic surgeons works.<br>(1: not at all, 2: rarely, 3: partly, 4: often, 5: always).<br>Why did you pick that number?<br>What do you think it takes to make the shared task of shoulder examinations work well?
Debriefing	Do you have anything you would like to share about you work relations with the orthopaedic surgeons?
