# Supplementary material for: Inter-professional agreement and collaboration between extended scope physiotherapists and orthopaedic surgeons in an orthopaedic outpatient shoulder clinic – a mixed methods study
Source: BMC Musculoskelet Disord. 2021 Jan 4;22:4. doi: 10.1186/s12891-020-03831-z (PMC7784386; doi:10.1186/s12891-020-03831-z)
Supplement: Supplementary file 3 — Additional file 3. Supplemental results. The file includes two supplemental tables: Distribution and number of diagnoses registered, and Agreement between ESPs and OSs on suggested treatment (three categories). [file 12891_2020_3831_MOESM3_ESM.docx]

**Additional file 3**

**Supplemental results**

*Supplemental Table 1. Distribution of diagnoses (n=69, unless otherwise stated)*

|  | **Diagnoses** | ESPs  Number (%) | OSs  Number (%) | Common decision  Number (%) |
| --- | --- | --- | --- | --- |
| Primary diagnosis* | Subacromial mpingement | 33 (48) | 19 (28) | 23 (33) |
|  | Rotator cuff injury | 11 (16) | 11 (16) | 11 (16) |
|  | Glenohumeral instability | 2 (3) | 2 (3) | 1 (1) |
|  | Glenohumeral osteoarthritis | 1 (1) | 1 (2) | 1 (1) |
|  | Adhesive capsulitis | 11 (16) | 12 (18) | 12 (17) |
|  | Scapula instability | 2 (3) | 7 (10) | 6 (9) |
|  | Fracture sequelae | 0 | 0 | 0 |
|  | Acromioclavicular joint disorder | 8 (12) | 14 (21) | 13 (19) |
|  | Non-shoulder related diagnosis | 1 (1) | 2 (3) | 2 (3) |
| Number of secondary diagnoses | 0 | 9 (13) | 15 (22) | 13 (19) |
|  | 1 | 32 (46) | 31 (45) | 38 (55) |
|  | 2 | 22 (32) | 20 (29) | 15 (22) |
|  | 3 | 6 (9) | 2 (3) | 3 (4) |
| Distribution of secondary diagnoses | Subacromial impingement | 24 (35) | 30 (44) | 27 (39) |
|  | Rotator cuff injury | 8 (12) | 5 (7) | 7 (10) |
|  | Glenohumeral instability | 6 (9) | 3 (4) | 5 (7) |
|  | Glenohumeral osteoarthritis | 3 (4) | 6 (9) | 2 (3) |
|  | Adhesive capsulitis | 3 (4) | 1 (1) | 2 (3) |
|  | Scapula instability | 16 (23) | 5 (7) | 6 (9) |
|  | Fracture sequelae | 3 (4) | 0 | 1 (1) |
|  | Acromioclavicular joint disorder | 25 (36) | 22 (32) | 20 (29) |
|  | Non-shoulder related diagnosis | 6 (9) | 5 (7) | 7 (10) |
| ESP: Extended scope physiotheraoist; OS: Orthopaedic surgeon  *ESP: n= 69, OS: n= 68, Common decision: n=69 | | | | |

*Supplemental Table 2.
Agreement between ESPs and OSs on suggested treatment (three categories) (n=68)*

|  | | **OS** | | | | Agreement  Number  (%) [95 % Cl] |
| --- | --- | --- | --- | --- | --- | --- |
|  |  | Possibly invasive | Physio- therapy | No intervention | Total | 60  (88) [81; 96] |
| **ESP** | Possibly invasive | 60 | 5 | 0 | 65 |  |
|  | Physiotherapy | 3 | 0 | 0 | 3 |  |
|  | No intervention | 0 | 0 | 0 | 0 |  |
|  | Total | 63 | 5 | 0 | 68 |  |

ESP: Extended scope physiotherapist; OS: Orthopaedic surgeon

Kappa =-0.06, Prevalence-adjusted and bias-adjusted kappa= 0.76, Prevalence index= -0.88, bias index= -0.03.
